# Revisiting the social determinants of health with explainable AI: a cross-country perspective

**DOI:** 10.1093/aje/kwaf205

**Published:** 2025-09-17

**Authors:** Jiani Yan

**Affiliations:** Leverhulme Centre for Demographic Science, University of Oxford, Oxford, United Kingdom; Department of Sociology, University of Oxford, Oxford, United Kingdom; Wolfson College, University of Oxford, Oxford, United Kingdom

**Keywords:** social determinants of health, cross-national comparisons, machine learning, explainable AI, predictive precision

## Abstract

In social science and epidemiological research, individual risk factors for mortality are often examined in isolation, while approaches that consider multiple risk factors simultaneously remain less common. Using the Health and Retirement Study in the United States, the Survey of Health, Ageing and Retirement in Europe, and the English Longitudinal Study of Ageing in the UK, we explore the predictability of death with machine learning and explainable AI algorithms, which integrate explanation and prediction simultaneously. Specifically, we extract information from all datasets in 7 health-related domains, including demographic, socioeconomic, psychology, social connections, childhood adversity, adulthood adversity, and health behaviors. Our self-devised algorithm reveals consistent domain-level patterns across datasets, with demography and socioeconomic factors being the most significant. However, at the individual risk-factor level, notable differences emerge, emphasizing the context-specific nature of certain predictors.

This article is part of a Special Collection on Cross-National Gerontology.

## Introduction

The contention that death is not merely biologically determined has increasingly been embraced by recent research.^1^ Specifically, social epidemiology emphasizes the distribution of social determinants of health.^2^ Manifold literature substantiates the significance of social determinants of health. Theoretically, studies such as Link and Phelan^3^ present the Fundamental Cause Theory, positing that health is associated with multiple social factors including wealth and education. Socioeconomic status, for example, influences health through various mechanisms such as access to nutritious food, adopting healthy lifestyles, and the availability of medical resources. This relationship is complicated and pervasive.^4^

Lorant et al.^5^ conducted a meta-analysis to assess the magnitude, shape and modifiers of the association between socioeconomic status and depression, finding compelling evidence for socioeconomic inequality in depression. Stafford and Marmot^6^ examined the impact of neighborhood socioeconomic status on individual health outcomes, revealing that poorer individuals who live in deprived neighborhoods experience the most significant negative health effects. Additional evidence has been accrued regarding other social factors such as social relationships,^7^ mental health,^8^ and childhood adversity.^9^

Nevertheless, social determinants of health are inherently multidisciplinary, necessitating a holistic study design. For example, the biopsychosocial model proposed by Engel^10^ underscores the interplay among social, biological, and psychological factors, where all of these factors dynamically interact with each other and continuously affect health outcomes. Moreover, intersectionality theory specifically highlights that disadvantages affecting life outcomes (including health) accumulate from multiple sources that are not independent of each other.^11^ Although the theory mainly focuses on interactions of individual identities, its emphasis on a holistic research framework facilitates understanding of the complicated, multidimensional nature of health inequalities.

While substantial evidence demonstrates the statistically significant association between theoretically proven risk factors and mortality, only a few studies have ventured beyond this explanatory framework to adopt an integrated approach alongside advanced methods. Puterman et al.^12^ compared the contribution of 57 economic, behavioral, social, and psychological factors in predicting death within the US context using multivariate Cox regressions, LASSO, and random forests. Similarly, Breen and Seltzer^13^ applied machine learning algorithms to predict life expectancy based on early adulthood socioeconomic and demographic information using US administrative data. In a different context, Vabalas et al.^14^ employed Finnish registry data to develop a deep-learning algorithm for modeling one-year mortality, incorporating over 8000 features from the domains of medical care, socioeconomic status, and demography. While these studies achieved varying levels of predictive performance, they are all limited to modeling within a single country’s perspective.

Given that health outcomes reflect the effects of health policy, medical conditions, and cultural differences,^15^ comparing the impact of established risk factors across different countries can provide valuable insights for improving health inequalities regionally. For example, Raghupathi and Raghupathi^16^ explored the association between adult education level, specifically upper secondary education, and cancer mortality in OECD countries by continent. While the association indicates that a higher proportion of upper secondary education correlates with fewer cancer deaths across all continents, the strength of this association varies, with North America exhibiting the strongest correlation.

Consequently, we propose a holistic approach to examine death, as one of the most definitive factors linked with health and its most notable downstream consequences. Here, “holistic” refers to a comprehensive, multidisciplinary research framework that incorporates a wealth of health-related information. However, holism should not be misconstrued as focusing solely on the quantity of risk factors. Instead, it ought to be conceived in terms of the relevance and utility of information to the research topic.^17^ Therefore, in our research, we elect to reexamine the risk factors from the work of Puterman et al.,^12^ which encompasses information from 7 different domains: demography, socioeconomics, psychology, social connections, childhood adversity, adulthood adversity, and health behaviors. This approach not only enhances the credibility of the results and potentially improves prediction accuracy, but also offers the opportunity to compare the relative contributions of factors from different domains. To foster cross-country comparisons, we employ data from three different data sources: the US Health and Retirement Study (HRS), the English Longitudinal Study of Ageing (ELSA), and the Survey of Health, Ageing and Retirement in Europe (SHARE). All of these are sampled from developed countries and are part of the Gateway to Global Ageing Data, which ensures a similar data structure, facilitating meaningful comparisons.

Methodologically, we adopt a predictive framework as most theories are developed based on internal processes, leaving their external predictive validity rarely tested. Additionally, considering the need to bridge explanation and prediction, we propose an innovative research paradigm to estimate the risk factors of death. This involves training a high-accuracy predictive model using advanced machine learning algorithms and deconstructing the results with explainable artificial intelligence methods, such as Shapley Values,^18^ to present an explainable framework. This approach remains comparatively unexplored, and it helps to unpack predictability and expose inequalities and biases residing in social systems by revealing the impact and direction of predictors, allowing further investigation, scientific discovery, and improved policy-making.

## Methods

### Data

The comprehensive design of the HRS, SHARE, and ELSA fosters a holistic investigation into risk factors influencing death probabilities. We have selected the HRS as the primary dataset for calibration. All risk factors are harmonized and comparable across different datasets (see Figure S1 for details of the sampling procedure and data sources for all datasets; and Figure S2 for data description and risk factor availability). The study focuses on individuals aged over 50 years, with summary information for the three main datasets presented in Table 1 (see Table S1 for combined datasets). See Tables S2 and S3 for the coverage of risk factors by dataset and domain, respectively.

**Table 1:** Overview of three mortality datasets.

**Dataset**	**HRS**	**SHARE**	**ELSA**
Age range, years	50-100	50-99	50-97
Female portion	0.592	0.545	0.553
Risk factor number	61	25	25
Sample size	13 210	17 818	8389
Death prevalence	0.316	0.214	0.169
Prediction window	2008-2019	2006-2021	2005-2012

**Figure 1:**
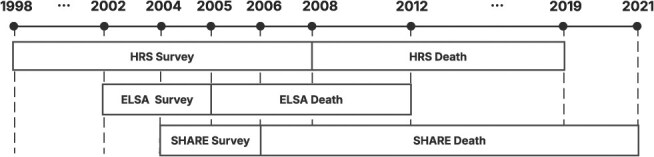
Sampling and death window of three datasets.

In this study, we define the outcome variable death as a binary indicator, where 1 denotes death and 0 indicates survival until the end of the prediction window. We aim to predict the probability of death within a specified future time frame. Each dataset employs distinct sampling and prediction windows, as illustrated in Figure 1. The “Survey” windows correspond to the periods during which data collection was conducted, while the “Death” windows represent the time frames over which mortality outcomes were recorded. For instance, in the HRS dataset, we utilize information collected between 1998 and 2008 to predict deaths occurring from 2008 to 2019. This framework reframes our research question as follows: Given an individual’s previously collected data, what is the probability of death within the next *X* years (where *X* varies by dataset)?

**Figure 2:**
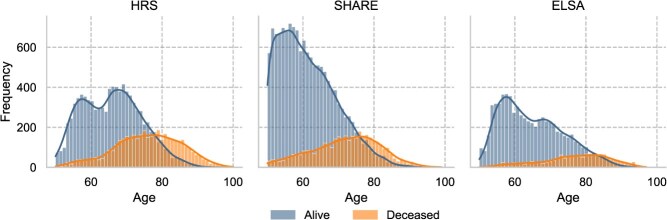
Age distribution by death status of three datasets.

We define death prevalence as the ratio $\frac{\mathrm{death\ counts\ within\ observation\ window}}{\mathrm{total\ observations\ within\ the\ sampling\ window}}$. For all individuals included in the analysis, either the exact death or a confirmed alive status is available. Figure 2 presents the age distributions by death status across the three datasets (Figure S3 provides age and gender decomposed death prevalence). Among them, ELSA exhibits the lowest death prevalence, which can be attributed to its relatively shorter prediction window.

### Statistical analysis

We estimate the predictability of elderly mortality using each of the three datasets individually as well as combined datasets, employing 12 classification machine learning algorithms. These algorithms include the Stochastic Gradient Descent classifier, K-nearest Neighbors classifier, Logistic regression, Decision Tree classifier, Support Vector Machines, Gaussian Naive Bayes, Adaptive Boosting Classifier, Bagging Classifier, Random Forest Classifier, Extra Trees Classifier, Light Gradient Boosting Machine (LightGBM), and Extreme Gradient Boosting Machine. We also adopt a state-of-the-art ensemble learning method, the Super Learner (SL), to estimate death predictability. Following the guidance of Bengio,^19^ all models are fitted multiple times with different seeds.

Model performances are assessed using several metrics: ROC-AUC, PR-AUC, R^2^, pseudo-*R*^2^, and the Inter-Model Vigorish (IMV) score. Higher values across all metrics indicate better performance. Among them, IMV offers a comparative assessment of binary classification performance across different models. ROC-AUC and PR-AUC measure the area under the receiver operating characteristic curve and the precision-recall curve, respectively. In the context of imbalanced datasets, PR-AUC is generally preferred over ROC-AUC, as the latter can be overly optimistic when the positive class is rare. Metric definitions can be found in SI: Evaluation Metrics. All datasets are split into conventional 7:3 train-test subsets, and training sets are used to fit models.

To ensure explainability, we select the algorithm with the best performance to investigate the source of predictability by calculating the Shapley Value for each risk factor. Shapley Values are designed to elucidate the importance of input variables within machine learning algorithms. The Shapley value (SHAP hereafter) of a risk factor *i* quantifies its marginal contribution to the predicted probability of death, relative to the model’s baseline prediction (ie, the mean predicted probability across all individuals). It captures how much *i* shifts the individual’s predicted probability from this average (see SI: Methodology for technical details). Since SHAP values can be both positive and negative, we primarily present the mean absolute SHAP for interpretation. Given the sensitivity involved in predicting subjective mortality, individual-level SHAP values will not be presented.

In addition to analyzing single risk factors, we devise a new leave-one-domain-out algorithm to estimate the domain-level importance in prediction accuracy. This algorithm iterates through all possible domain combinations (127 combinations with seven domains) and calculates the marginal contribution of each specific domain to the evaluation metric. The final domain importance is the mean of all marginal contributions across every possible combination. This approach provides a comprehensive comparison of risk factors at an aggregate level.

## Results

### Death predictability

Table 2 presents the average model evaluation metrics across 10 different seeds for the 3 individual datasets and the combined datasets, respectively. The standard errors are reported in parentheses. Higher values for all metrics indicate better out-of-sample predictive performance. BM stands for the benchmark model of Logistic Regression trained with age and gender only, following the practice in Vabalas et al.^14^ As most of our samples are uneven regarding death outcomes—with test set in-sample prevalence ranging from 0.169 in ELSA to 0.315 in HRS—we use the PR-AUC score as the primary machine learning evaluation metric. We find that death is generally highly predictable across all models and datasets, as the PR-AUC scores are more than double the in-sample prevalence, which represents the probability of random guessing and serves as the interpretation baseline of PR-AUC. Predictions made using HRS and combined datasets demonstrate better performance, as both dataset sizes and in-sample prevalence are significantly larger compared to SHARE and ELSA. Surprisingly, the ensemble learner SL performs worse than LightGBM in all scenarios, signaling that LightGBM achieves overwhelmingly strong predictive performance and thus is not suitable for averaging with other algorithms in this case.

**Table 2:** Average model performance for death prediction across 10 random seeds.

	**HRS**			**SHARE**			**ELSA**		
**Metrics**	**SL**	**LightGBM**	**BM**	**SL**	**LightGBM**	**BM**	**SL**	**LightGBM**	**BM**
IMV	0.196	0.202	0.175	0.094	0.098	0.093	0.061	0.069	0.067
	(0.003)	(0.003)	(0.046)	(0.002)	(0.002)	(0.002)	(0.002)	(0.002)	(0.001)
ROC-AUC	0.816	0.820	0.790	0.812	0.815	0.806	0.824	0.832	0.818
	(0.001)	(0.001)	(0.001)	(0.002)	(0.002)	(0.003)	(0.004)	(0.003)	(0.003)
PR-AUC	0.695	0.698	0.675	0.575	0.586	0.566	0.508	0.533	0.529
	(0.002)	(0.003)	(0.003)	(0.005)	(0.005)	(0.005)	(0.009)	(0.007)	(0.007)
*R* ^2^	0.282	0.287	0.246	0.246	0.253	0.239	0.212	0.240	0.231
	(0.002)	(0.002)	(0.002)	(0.004)	(0.005)	(0.004)	(0.009)	(0.007)	(0.006)
Pseudo *R*^2^	0.282	0.287	0.246	0.246	0.253	0.239	0.212	0.241	0.231
	(0.002)	(0.002)	(0.002)	(0.004)	(0.005)	(0.004)	(0.009)	(0.007)	(0.006)
IP	0.315	0.315	0.315	0.214	0.214	0.214	0.169	0.169	0.169
	**HRS + SHARE**			**HRS + ELSA**			**SHARE + ELSA**		
**Metrics**	**SL**	**LightGBM**	**BM**	**SL**	**LightGBM**	**BM**	**SL**	**LightGBM**	**BM**
IMV	0.136	0.138	0.124	0.139	0.142	0.122	0.082	0.086	0.076
	(0.002)	(0.001)	(0.001)	(0.002)	(0.002)	(0.002)	(0.001)	(0.001)	(0.001)
ROC-AUC	0.821	0.823	0.804	0.823	0.825	0.796	0.811	0.816	0.798
	(0.001)	(0.001)	(0.001)	(0.001)	(0.001)	(0.001)	(0.002)	(0.002)	(0.002)
PR-AUC	0.642	0.647	0.621	0.646	0.650	0.618	0.548	0.561	0.523
	(0.003)	(0.003)	(0.003)	(0.002)	(0.003)	(0.003)	(0.004)	(0.003)	(0.004)
*R* ^2^	0.277	0.281	0.250	0.276	0.280	0.240	0.233	0.246	0.213
	(0.002)	(0.002)	(0.002)	(0.002)	(0.002)	(0.002)	(0.003)	(0.003)	(0.003)
Pseudo *R*^2^	0.277	0.281	0.250	0.277	0.280	0.240	0.234	0.246	0.213
	(0.002)	(0.002)	(0.002)	(0.002)	(0.002)	(0.002)	(0.003)	(0.003)	(0.003)
IP	0.258	0.258	0.258	0.259	0.259	0.259	0.200	0.200	0.200
	**HRS + SHARE + ELSA**								
**Metrics**	**SL**			**LightGBM**			**BM**		
IMV	0.118			0.121			0.108		
	(0.001)			(0.001)			(0.001)		
ROC-AUC	0.817			0.821			0.801		
	(0.001)			(0.001)			(0.001)		
PR-AUC	0.616			0.624			0.592		
	(0.001)			(0.002)			(0.002)		
*R* ^2^	0.264			0.271			0.238		
	(0.002)			(0.002)			(0.002)		
Pseudo *R*^2^	0.264			0.271			0.238		
	(0.002)			(0.002)			(0.002)		
IP	0.239			0.239			0.239		

Note: Standard errors are shown in parentheses.

### Cross sectional similarities: domain contribution

**Figure 3:**
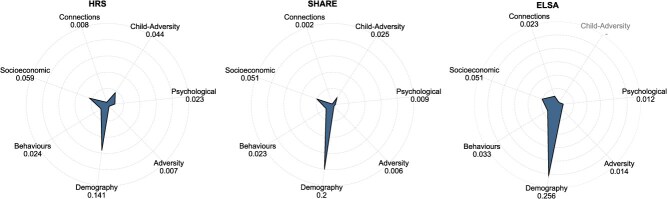
Domain-level marginal prediction contribution of all datasets. It presents the domain-level risk factors’ marginal PR-AUC score contribution. LightGBM is used for all model fitting procedures. Consistent patterns exist in HRS, SHARE, and ELSA.

Figure 3 illustrates the domain-level contribution of risk factors in improving the PR-AUC score using the LightGBM model for each dataset separately. The HRS and SHARE datasets encompass predictors from all 7 domains, while ELSA includes 6 domains. The number of predictors within each domain varies across datasets.

A consistent pattern emerges in all datasets, highlighting the demographic and socioeconomic domains as the most influential in enhancing the PR-AUC score, thereby improving the precision and accuracy of predictions. This finding aligns with the Fundamental Cause Theory proposed by Link and Phelan,^3^ which posits that socioeconomic status exerts a persistent influence on health through multiple mechanisms, playing a fundamental role in health disparities. The demographic domain, encompassing key variables such as age and gender, consistently exhibits a substantial contribution across all datasets. This demographic-socioeconomic pattern also exists in the combined datasets, as shown in Figure S4.

It is important to acknowledge that the model’s predictive performance is inherently shaped by the amount of relevant information presented in the data. For instance, in the HRS dataset, the psychological domain includes the largest number of risk factors, which partially explains its substantial contribution to domain-level predictive performance. However, the number of predictors alone does not fully determine domain importance. For example, in SHARE, the demographic and psychological domains contain 3 and 6 factors, respectively, yet the demographic domain’s contribution is more than 20 times greater than that of the psychological domain.

In ELSA, the absence of the childhood-adversity domain may result in an incomplete assessment of risk factor importance, particularly in capturing the marginal impact associated with the psychological and social connection domain. As demonstrated by Kessler,^20^ childhood adversities have a significant impact on adult mental disorders across 21 countries. Additionally, the distinct pattern of domain importance observed in ELSA may, in part, be attributed to the short prediction window, which restricts the dataset’s capacity to capture variable importance beyond the demographic domain.

### Cross sectional dissimilarities: risk factor importance

Beyond assessing risk factor importance from a model performance perspective, we calculate SHAP values for a representative model from each dataset to decompose predicted death probabilities. Our objective is to provide insights into variable importance within robust predictive frameworks, aligning with the interpretative nature of SHAP values. Notably, SHAP values can accurately reflect factor importance only when the predictive algorithm is reliable. To mitigate the risk of over-interpreting unstable results, we present only those risk factors with stable SHAP values (ie, their mean $\mid \mathrm{SHAP}\mid$  > 0.1) that rank among the top tier in risk comparisons.

**Figure 4:**
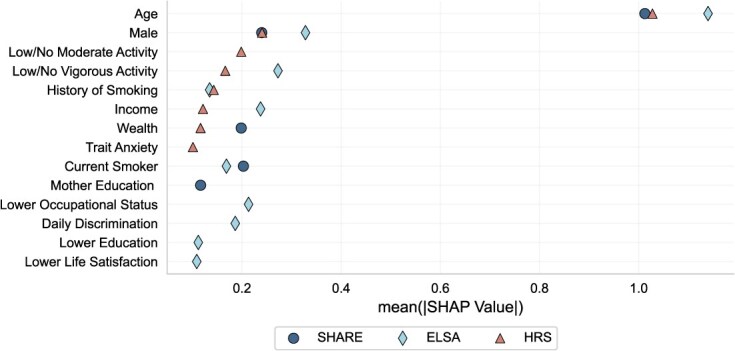
Risk factor importance of three datasets. Risk factors with mean $\mid \mathrm{SHAP}\mid$ > 0.1 from the predictions of HRS, SHARE, and ELSA are displayed in the figure. Mean $\mid \mathrm{SHAP}\mid$ values were calculated across the entire dataset (train + test) to comprehensively reflect the overall feature contribution, capturing both model fitting and generalization behaviors. Risk factor ranks are calibrated based on their order in HRS. The ranks and factors vary across different predictions. Common risk factors across the three predictions are age and gender.

Figure 4 highlights the key differences among the datasets, which primarily stem from three aspects: the specific risk factors identified, their relative rankings, and their levels of importance. In HRS, the most influential risk factors (ranked from highest to lowest) are age, gender, low/no moderate activity, low/no vigorous activity, history of smoking, income, wealth, and trait anxiety. In SHARE, the most critical predictors include age, gender, current smoking status, wealth, and maternal education. In ELSA, the top-ranked risk factors encompass age, gender, low/no vigorous activity, income, lower occupational status, daily discrimination, current smoking, history of smoking, lower education, and lower life satisfaction. Notably, ELSA exhibits the broadest range of significant risk factors, spanning nearly all domains except the psychological domain.

**Figure 5:**
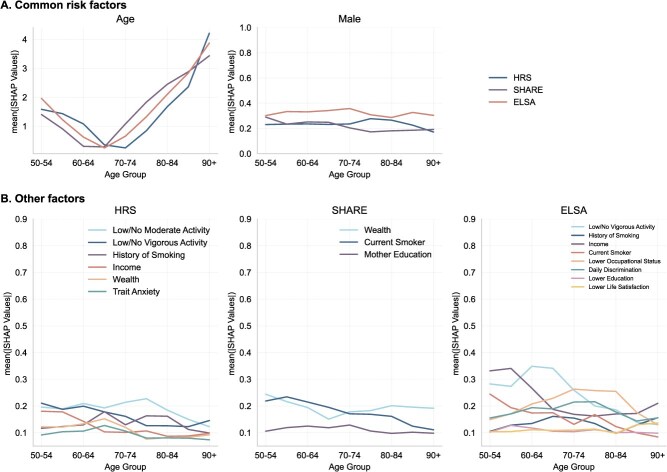
Age-specific risk factor importance. Subfigure A shows the age-based importance of the three common variables, represented by the mean $\mid \mathrm{SHAP}\mid$ values. Subfigure B shows the importance trend of the top variables having overall mean $\mid \mathrm{SHAP}\mid$ values >0.1 based on different datasets.

Among these top-ranked risk factors, only age and gender consistently emerge across all datasets. Age demonstrates a clear gradient effect in all cases. Gender, despite exhibiting different levels of importance across datasets, maintains the second-most important rank among all datasets and a relatively stable trend across both age groups and datasets (see Figure 5). Income and wealth collectively emerge as the most influential socioeconomic risk factors across all datasets. In ELSA, income ranks as the forth most significant predictor, whereas its impact—along with wealth—is less pronounced in SHARE and HRS.

Some risk factors appear to be context-dependent. Beyond age and gender, six additional risk factors are present across all datasets in Figure 4: history of smoking, wealth, low/no vigorous activity, current smoking, lower education, and lower occupational status. However, the importance of these factors varies by context. In the US context, current smoking status, lower education, and lower occupational status do not rank among the most important factors (mean $\mid \mathrm{SHAP}\mid$ > 0.1). Conversely, in the European datasets (SHARE), history of smoking, low/no vigorous activity, and lower occupational status are not among the most influential predictors.

Furthermore, even among common risk factors, their relative importance varies. For example, income is a stronger predictor in ELSA than in HRS, which may be attributed to ELSA’s exclusive reliance on income rather than both income and wealth. Similarly, low/no vigorous activity plays a more significant role in ELSA compared to HRS, despite their similar mean values of approximately 0.7. In terms of smoking status, SHARE stands out by identifying only current smoking as a significant risk factor, whereas history of smoking is more relevant in the other datasets. This distinction is likely due to the substantially higher prevalence of current smokers in SHARE (mean value: 0.48) compared to HRS (0.12) and ELSA (0.18).

Given our focus on predicting mortality outcomes in the elderly population, we further stratify the mean absolute SHAP values ($\mid \mathrm{SHAP}\mid$) by age groups within each dataset, as shown in Figure 5. This stratification helps identify age-related gradients in feature importance and enables more nuanced cross-sectional comparisons across datasets.

Among the common predictors, age exhibits a consistent “U-” or “V-”shaped pattern in all 3 datasets. Notably, the lowest $\mid \mathrm{SHAP}\mid$ values occur within the 60-75 age range, which corresponds approximately to the mean age at death in each dataset. This indicates that age contributes less to mortality prediction near the average death age, and becomes more influential for individuals at both younger and older extremes. Details about the SHAP interpretation of age can be found in SI: SHAP explanation of age.

Although age remains the most influential risk factor, its predictive importance in identifying mortality risk diminishes around this mean death age. Instead, age becomes more critical in predicting mortality among the oldest-old (those aged older than 80 years), where the mean $\mid \mathrm{SHAP}\mid$ values exceed 1.5 in all datasets. Gender, by comparison, shows a relatively stable contribution to mortality prediction across age groups. The consistent prominence of age and gender, both demographic variables, highlights the dominant role of the demographic domain in predicting mortality risk in older populations across diverse settings.

Panel B further illustrates age-specific trends in variable importance across different risk factors and datasets. In the US dataset, while most risk factors exhibit moderate fluctuations across age groups, income, low/no vigorous activity, and trait anxiety demonstrate a mild downward trend with increasing age. In SHARE, current smoking status declines in importance as a predictor of mortality after age 60. The ELSA dataset displays the highest degree of variation, which may be partly attributed to the SHAP decomposition algorithm, where variable importance is computed based on predicted values. Income follows a “V-shaped” pattern, with higher importance among both younger and the oldest age groups. In contrast, lower occupational status exhibits a peak around ages 70-74 years, following an inverted “U-shape” across different age groups.

Despite their overall importance, health behavior risk factors—such as smoking history, current smoking status, and low or no engagement in vigorous physical activity—exhibit a declining trend in predictive importance with advancing age across all datasets. This trend may be attributed to the general decline in individuals’ ability to engage in physical activity as they age, which reduces the variability and predictive power of this factor in older age groups.

## Discussion

In this study, we first address the question of how well mortality can be predicted using social determinants by selecting the best-performing model among multiple machine learning algorithms. Utilizing information from multiple domains, we achieve high predictive performance across all datasets: in HRS, SHARE, and ELSA, the highest ROC-AUC scores are 0.820, 0.815, and 0.832, respectively, while PR-AUC scores reach 0.698, 0.586, and 0.533, respectively. Notably, the predictive performance of ELSA improves significantly when integrated with other datasets, particularly in combination with HRS (PR-AUC = 0.650).

At the domain level, we develop a leave-one-out algorithm to assess the marginal contribution of each domain, averaging its predictive performance impact across all possible combinations. Despite variations in the number of risk factors across domains, consistent patterns emerge. In all datasets, demographic and socioeconomic factors are the most influential predictors. In HRS and SHARE, the child-adversity domain ranks third in importance. Health behaviors domain holds this position in ELSA. It also suggests that while key domains influencing mortality are relatively stable across countries, their relative importance may vary. These findings align with previous research indicating that while individual domains may have limited predictive power in isolation,^13^ their combined effects are critical for capturing mortality risk.

Dissimilarities are largely discovered in the decomposition of prediction using SHAP values. This decomposition enables us to identify the most influential individual risk factors for mortality prediction within each dataset, reflecting the unique characteristics and contextual influences of different populations. Age and gender consistently emerge as critical predictors across all datasets, underscoring the foundational role of demographic factors in mortality modeling among aging populations. This finding aligns with existing literature. For example, Aida et al.^21^ shows a large-scale change of survival days in both ELSA and the Japan Gerontological Evaluation Study with increasing age. Lantz et al.^22^ demonstrates significant survival differences between females and males among US adults.

However, beyond these commonalities, different datasets prioritize different risk factors. In HRS, the top-tier risk factors (mean $\mid \mathrm{SHAP}\mid$ > 0.1) highlight the significance of socioeconomic status, health behaviors, and mental health within the US context. In SHARE, socioeconomic conditions, health behaviors, and childhood adversity emerge as key predictors, aligning with domain-level analyses. ELSA exhibits the broadest set of high-importance risk factors, encompassing multiple domains, which may be attributable to its inferior model performance and incomplete coverage of risk factors at the domain level.

Notable regional variations exist. For instance, current smoking status is highly influential in SHARE but not in HRS. Conversely, factors such as history of smoking and low/no vigorous activity play a more prominent role in HRS but not in the European datasets. Moreover, while ELSA shares many high-importance risk factors with both HRS and SHARE, it uniquely emphasizes lower occupational status and lower education as key predictors. These differences highlight the necessity of context-specific approaches to mortality risk assessment, reinforcing the importance of integrating both generalized and localized perspectives in demographic and health research.

Figure 5 also shows a general downward trend in health behavior factors, suggesting a diminishing relative contribution of these factors in predicting mortality among the oldest-old, though they remain significant (mean $\mid \mathrm{SHAP}\mid$ > 0.1). While extensive research has established the link between health behavior and mortality, particularly all-cause mortality (see Duncan et al.^23^ for an example), few studies have systematically decomposed their importance across different age groups. This decline may be attributed to the overriding influence of age in late-life mortality risk or to physical limitations among the oldest-old, which may render certain health behaviors, such as vigorous physical activity, less relevant in distinguishing mortality risk within this group.

Despite the strengths of this study, several limitations must be acknowledged. First, discrepancies in data collection methodologies across datasets result in imperfect alignment of risk factors. This mismatch reduces the number of directly comparable variables when integrating datasets, thereby constraining cross-contextual comparisons (see Figure S2). For instance, only 25 out of 61 risk factors in HRS have direct counterparts in SHARE.

Second, our analysis is limited to mortality prediction within high-income countries—the U.S., Europe, and the U.K. While prioritizing variable consistency enables more robust modeling, the exclusion of datasets from low- and middle-income countries restricts the generalizability of our findings. Incorporating data from comparable large-scale studies, such as the China Health and Retirement Longitudinal Study and the Longitudinal Aging Study in India, would enable a more comprehensive examination of global health disparities.

Finally, the exclusion of biological and genetic variables due to data limitations constrains the comprehensiveness of our models. Integrating biomarkers and genetic data could enhance predictive accuracy and provide deeper insights into the complex interplay between biological and social determinants of health. As such data become increasingly available, future studies should seek to incorporate these dimensions to refine mortality prediction models further.

## Supplementary Material

Web_Material_kwaf205

## Data Availability

Data should be retrieved from the original data providers: Health and Retirement Study; English Longitudinal Study of Aging; and the Survey of Health, Aging and Retirement in Europe.
