# Supplementary material for: Revisiting the social determinants of health with explainable AI: a cross-country perspective
Source: Am J Epidemiol. 2025 Sep 17;195(3):681–8. doi: 10.1093/aje/kwaf205 (PMC13017747; doi:10.1093/aje/kwaf205)
Supplement: Web_Material_kwaf205 [file web_material_kwaf205.pdf]

Revisiting the social determinants of health with explainable AI: a cross-country perspective
Supplementary Information

Jiani Yan

Contents

Contents **1**

1 Methodology **2**
- 1.1 Super Learner 2
- 1.2 LightGBM 3
- 1.3 Shapley Values 3

2 Evaluation Metrics **4**

3 SHAP Explanation of Age **5**

4 Model Performance of Combined Datasets **5**

5 Tables **7**
- Table S1 - Death Window and Prevalence of Combined Datasets 7
- Table S2 - Risk Factors by Dataset and Domain 8
- Table S3 - Risk Factors of Combined Datasets 9

6 Figures **10**
- Figure S1 - Data pruning process illustration for all datasets 10
- Figure S2 - Risk factor inclusion and exclusion, mean value and standard deviation by dataset 11
- Figure S3 - Death prevalence by age and gender for all datasets. 12
- Figure S4 - Domain contribution for all combined datasets. 12
- Figure S5 - Mean SHAP Values of Age Across Different Age Groups. 13

1 Methodology

1.1 Super Learner

Developed by Laan, Polley, and Hubbard [1], a Super Learner (SL) is an ensemble algorithm that combines multiple different machine learning models together to produce a prediction that performs better than a single model. Ensemble models have long been discussed and explored; normally scientists choose to ensemble models either by averaging or applying some pre-defined weights, usually generated from the model performance. However, Laan, Polley, and Hubbard [1] has devised the SL, which aims to use a learning algorithm to cross-validate and find the optimal weights to combine a collection of predictive models.

To facilitate understanding, we divide the whole process into two parts: the cross-validation stage and the ensemble learning stage. We start illustrating by defining a set of m base predictive algorithms as $g_1, g_2, g_3, ..., g_m$, each representing a single candidate learner applied in the SL. In our case, as we aim to predict a binary outcome, we choose a set of classifiers including a stochastic gradient descent classifier, K-neighbors Classifier, Logistic Regression, Decision Tree classifier, Support Vector Machines, Gaussian Naive Bayesian, Adaptive Boosting Classifier, Bagging Classifier, Random Forest Classifier, Extra Trees Classifier, Light Gradient Boosting Machine Classifier, eXtreme Gradient Boosting Machine Classifier as the basic learners.

In the cross-validation stage, the ultimate goal is to construct and retrieve the performance of each learner in the training set. Specifically, the training set will be split into v mutual exclusive and nearly equal size sub-sets where candidate learners will be cross-validates on each of the v splits. In other words, further by splitting the original training set into v splits, selecting one of them v_i as a sub-validation set and the rest as sub-training sets ($v-1$ splits), every learner will be trained on the sub-training set and validated on the sub-validation set, generating a corresponding prediction of v_i. Repeating this procedure v times (validation set v_i, $i = 1, ..., v$) to generate a complete list of v predictions. Therefore, after v times of repeating validation on m single learners, we will generate a new space of possible outcomes Z, which has the same number of rows as the training set and m columns.

Then we move to the ensemble learning stage where the main goal is to train a user-defined learning algorithm $\tilde{\Psi}$ that minimises the squared error loss function. Specifically, $\tilde{\Psi}$ is designed to estimate the regression $E(Y|Z)$, where Y is the real label of the training set. The $\tilde{\Psi}$ can also be defined as the minimum cross-validated risk predictor as it minimises the cross-validated risk generated in the last step. Lastly, the fitted $\tilde{\Psi}$ will be applied to the single learners that are trained on the whole training set. Based on the data P_n and value X, the SL is given by

$$\hat{\Psi}(P_n)(X) \equiv \hat{\Psi}^*(P_n)\left(\hat{\Psi}_j(P_n)(X), j = 1, \ldots, m\right) \tag{1}$$

where $\hat{\Psi}$ is the mapping function ensembles the m learners from their risks obtained from the cross-validate step and $\hat{\Psi}^*(P_n)$ is the actual obtained predictor from the mapping function. In other words, in a SL, the Y for a value X is obtained by evaluating the predictor $\hat{\Psi}^*(P_n)$ at the J predicted values at X of the m candidate learners. In our case, we select LightGBM as the ensemble mapping learner.

1.2 LightGBM

As LightGBM is selected as a benchmark method, we briefly review its mechanism. Developed by Ke et al. [2], LightGBM is a cutting-edge variant of gradient boosting machine (GBM). In essence, it is a powerful ensemble learning method that aggregates multiple weak classifiers together to form a single learner in the manner of reducing errors along the gradient direction. It can be described with equations as follows: suppose we have T regressions trees $f_t(X)$ and the ultimate predicted value is generated through an integrated regression tree of the T regressions, i.e. $F_t(x_i) = \sum_{t=1}^{T} f_t(X)$. The integration is conducted through minimising a specific loss function $\sum_i l\left(\hat{y}_i, y_i\right)$ where the y_i is the true value of death occurrence and $\hat{y}_i$ is the predicted value. We choose the log loss here as our outcome variable is binary. During integration at the tth step, the LightGBM method aims to minimise the value of following equation:

$$\min \Gamma^{(t)} = \min \sum_{i=1}^{n} l\left(y_i, F_{t-1}\left(x_i\right) + f_t\left(x_i\right)\right) \tag{2}$$

The minimisation task is undertaken by Newton's method as follows:

$$\Gamma_t \cong \sum_{i=1}^{n} \left(g_i f_t\left(x_i\right) + \frac{1}{2} h_i f_t^2\left(x_i\right) \right) \tag{3}$$

where again, $g_i = \partial_{\hat{y}_i} L\left(\hat{y}_i, y_i\right)$ is the first-order gradient function of the loss function and $h_i = \partial_{\hat{y}_i}^2 L\left(\hat{y}_i, y_i\right)$ is the second-order. Compared to other GBMs, Light GBM is novel in two unique techniques to tackle big data: Gradient-based One-Side Sampling (GOSS) and Exclusive Feature Bundling (EFB). With GOSS, it can reduce the sample size possessing small gradients as it is defined in information gains that only larger gradients contribute to higher information gain. EFB is designed to reduce the sparsity of the feature space through bundling features that are exclusive and rarely having non-zero values at the same time. Combining the two techniques together, the LightGBM has become one of the most efficient and scaleable boosting decision tree methods.

1.3 Shapley Values

Shapley Values are designed to unravel the input variable importance within machine learning algorithms. For a single feature i, the classical Shapley Value is obtained through the following formula:

$$\phi_i = \sum_{S \subseteq N \setminus \{i\}} \frac{|S|!(M - |S| - 1)!}{M!} \left[f_x(S \cup \{i\}) - f_x(S)\right] \tag{4}$$

where S is a non-zero subset of the whole input features. M and N are the total number and the whole set of input features respectively (see Table 1 and Table S1 for the information for all datasets). $f_x(S)$ is the prediction function for feature in set S such as LightGBM. Under such a definition, the Shapley Value is a decomposition method that calculates the weighted difference between all possible subsets of the input features with and without the specific input feature i. Following the definition, Lundberg, Erion, and Lee [3] proposed the following equation to calculate Shapley Values:

$$f_x(S) = f(h_x(z')) = E[f(x)|x_s] \tag{5}$$

where $h_x(z')$ is a mapping function that maps between the original function and the left-out features (z', in a binary manner by setting $z'_i = 1$ or 0). $E[f(x)|x_s]$ is the expected function value conditioned on the subset S. The resulting Shapley Value will be called SHAP hereafter. In our research, the SHAP value for a risk factor i stands for the log odds being predicted as dead, which is the marginal contribution of i on the death prediction. A positive SHAP indicates a higher possibility of being predicted as dead and a negative value indicates a higher possibility of being predicted as being alive. As SHAP can be both positive and negative, to interpret SHAP, we adopt the mean absolute SHAP. Note that, unlike conventional regressions where a feature will only have one uniform 'β' presenting its contribution, SHAP is calculated at the individual level and therefore a distribution of i will be obtained. Appreciating the essence of predicting frailty in this research and to avoid interpretability at the single-factor level, the individual-level SHAP values will not be presented.

2 Evaluation Metrics

In this work, we will evaluate the out-of-sample predictive performance with two broad categories of evaluation metrics: conventional social science metrics and machine learning evaluation metrics. The Pseudo R^2 is calculated to provide conventional insights into the model goodness of fit, providing the following functions:

$$\text{Pseudo } \boldsymbol{R}^2 = 1 - \frac{\sum (y_i - \pi_i)^2}{\sum (y_i - \bar{y}_{test})^2} \tag{6}$$

where y_i stands for the real death situation of individual i (0 or 1) and π_i is the corresponding predicted death probability. $\bar{y_{test}}$ and $\bar{y_{train}}$ are the death prevalence of the test set and train set.

We will then use the classic PR-AUC score and the innovative Inter-Model Vigorish (IMV) score as machine learning evaluation metrics. The PR-AUC score is the Area Under the Curve of Precision and Recall, which emphasises the harmonic mean of precision and recall. It takes precision ($\frac{\text{TP}}{\text{TP+FP}}$) as the y-axis value, measuring the number of true positive observations against the total number of observations predicted as positive. In the x-axis, the recall rate ($\frac{\text{TP}}{\text{TP+FN}}$) measures the proportion of true positive in the real positive observations. One of the benefits of PR-AUC is that it focuses on the positive labels (death in our study) and therefore avoids a high score if the sample is unevenly distributed. As most of our samples have a very small portion of death (True) labels, PR-AUC is a better choice than the more prevalent ROC-AUC score, as ROC-AUC can easily gain a higher score if we randomly assign a False label to all samples. Using the PR-AUC grants us a more comprehensive understanding of our models. A higher PR-AUC is preferred as a better performance of the prediction, whereas in-sample prevalence (IP, True label portion of the training set) is viewed as a benchmark to interpret the PR-AUC score, any value higher than the IP is a sign of a 'good score'.

The IMV is a portable metric designed by Domingue et al. [4, 5] to understand the goodness of fit in the case of a binary outcome. We use the score as it's a comparable score between models and datasets, where our analysis involves evaluating the performance of four different datasets. To compute the IMV score, we start by establishing two binary predictive systems,

one of which is the baseline and the other one is the enhanced system. The enhanced system contains 'additional' information that is blind to the baseline system.

Recall that the entropy is defined as $-1(w \log w + (1-w)\log(1-w))$, to compute IMV, we aim to calculate the probability ω_0 and ω_1 that identifies the entropy of the baseline and enhanced system respectively that fulfils the following function:

$$\omega \log(\omega) + (1-\omega)\log(1-\omega) = \log(A) \tag{7}$$

where A is the geometric mean of the likelihood of the selected system, defined as:

$$A = \left(\prod_{i=1}^{n} L_i\right)^{\frac{1}{n}} = \left(\prod_{i=1}^{n} p_i^{y_i}\,(1-p_i)^{1-y_i}\right)^{\frac{1}{n}} \tag{8}$$

here p_i is the predicted death probability of individual i and y_i is the true death label of the corresponding individual. The ω_0 and ω_1 are calculated by minimising the difference between the entropy definition and $\log(A)$:

$$\min |\omega \times \log(\omega)| + (1-\omega) \times \log(1-\omega) - \log(A) \tag{9}$$

and the IMV score is simply the relative ratio of the two probabilities:

$$\text{IMV} = \frac{w_1 - w_0}{w_0} \tag{10}$$

It reflects the extent the enhanced system (usually the fitted models) outperforms the benchmark system (usually a simple model). We select the in-sample prevalence as w_0, corresponding to the PR-AUC score described above.

3 SHAP Explanation of Age

To further interpret the 'V-' or 'U-' pattern of age, we note that age is the only feature whose mean SHAP values (not just absolute values) cross the zero line. As shown in Figure S5, the original SHAP values for age exhibit a steadily increasing trend: younger participants have negative SHAP values, while older participants have positive ones. Since SHAP values represent the marginal contribution of a feature to the model output relative to the mean prediction, a negative SHAP value for age implies a lower predicted probability of death compared to the dataset average, and vice versa. Therefore, the observed 'U-' or 'V-' shaped pattern in |SHAP| values can be understood as reflecting the non-linear influence of age. Age becomes more important for individuals who deviate substantially from the mean age at death—either because they are significantly younger (indicating low risk) or significantly older (indicating high risk).

4 Model Performance of Combined Datasets

From the comparison of data performance, we can observe that combining a single dataset with another dataset that has better predictive performance improve the overall predictive capability. For example, any combination of ELSA with HRS and SHARE significantly enhances

its performance. Specifically, the PR-AUC increases from 0.533 for ELSA alone to 0.650 for HRS+ELSA and 0.624 for HRS+SHARE+ELSA. For ELSA, the primary contributors to this performance improvement are the in-sample prevalence (increasing from 0.169 to 0.259 and 0.239) and the sample size (increasing from 8,389 to 21,599 and 39,417). These two effects even outweigh the reduction in the number of risk factors in SHARE+ELSA, where the model still shows improvement despite a reduction of 13 risk factors. Using the combined dataset, we estimate the importance of exploring how variations in the scale of sample size and the number of risk factors affect model performance. A similar conclusion could be drawn from SHARE where combining a dataset with better performance could achieve higher scores: both SHARE+HRS and SHARE+HRS+ELSA have higher performance compared to the model trained on SHARE alone, where HRS+SHARE is better than the HRS+SHARE+ELSA model.

Conversely, combining a dataset with worse predictive performance slightly deteriorates the combined scores. For instance, the PR-AUC score of SHARE+ELSA is lower than that of SHARE alone. In this case, both the number of risk factors (decreasing from 25 to 13) and the in-sample prevalence (declining from 0.214 to 0.200) decrease, while only the sample size increases (from 17,818 to 26,207). Another example is the HRS dataset, which exhibits a slight overall negative impact due to the reduction in risk factors and in-sample prevalence, despite an increase in sample size.

5 Tables

Table S1: Death Window and Prevalence of Combined Datasets

Dataset	HRS+ELSA	HRS+SHARE	SHARE + ELSA	All
Age range	50-100	50-100	50-99	50-100
Female portion	0.577	0.565	0.548	0.563
Risk factor number	25	25	13	13
Sample size	21599	31028	26207	39417
Death prevalence	0.259	0.258	0.200	0.239
prediction window	2005 - 2019	2006 - 2021	2005 - 2021	2005 - 2021

Note that there are 13 death cases of ELSA from 2004, which happened after data collection.

Table S2: Risk Factors by Dataset and Domain

Dataset	Domain	Count	Variables
HRS	Demography	5	Male, Black, Age, Foreign Born, Hispanic
HRS	Child-Adversity	7	Father Education , Father was Unemployed in Childhood, Relocated Homes in Childhood, Childhood Psychosocial Adversities, Family Received Financial Help in Childhood, Mother Education , Father Occupational Status
HRS	Socioeconomic	13	History of Food Insecurity, History of Unemployment, Lower Neighborhood Safety, Lower Neighborhood Cohesion, History of Food Stamps, History of Medicaid, History of Renting, Wealth, Lower Education, Lower Occupational Status, Income, Neighborhood Disorder, Recent Financial Difficulties
HRS	Behaviours	6	History of Smoking, Alcohol Abuse, Sleep Problems, Low/No Vigorous Activity, Low/No Moderate Activity, Current Smoker
HRS	Adversity	3	Major Discrimination, Daily Discrimination, Adulthood Psychosocial Adversity
HRS	Connections	7	History of Divorce, Lower Positive Interactions with Family, Negative Interactions with Family, Lower Positive Interactions with Children, Negative Interactions with Children, Negative Interactions with Friends, Never Married
HRS	Psychological	20	Lower Purpose in Life, Lower Sense of Mastery, Pessimism, Lower Conscientiousness, Lower Neuroticism, Negative Affectivity, Perceptions of Obstacles, Lower Extroversion, Lower Optimism, Lower Openness to Experiences, Loneliness, Hopelessness, Cynical Hostility, Anger In, Anger Out, Lower Religiosity, Lower Life Satisfaction, Lower Agreeableness , Trait Anxiety, Lower Positive Affectivity
SHARE	Demography	3	Male, Age, Foreign Born
SHARE	Child-Adversity	3	Father Education , Mother Education , Father Occupational Status
SHARE	Socioeconomic	5	History of Unemployment, History of Renting, Wealth, Lower Education, Lower Occupational Status
SHARE	Behaviours	5	History of Smoking, Sleep Problems, Low/No Vigorous Activity, Low/No Moderate Activity, Current Smoker
SHARE	Adversity	1	Adulthood Psychosocial Adversity
SHARE	Connections	2	History of Divorce, Never Married
SHARE	Psychological	6	Pessimism, Negative Affectivity, Perceptions of Obstacles, Lower Optimism, Hopelessness, Lower Positive Affectivity
ELSA	Demography	3	Male, Age, Foreign Born
ELSA	Socioeconomic	8	History of Unemployment, Lower Neighborhood Cohesion, History of Renting, Wealth, Lower Education, Lower Occupational Status, Income, Neighborhood Disorder
ELSA	Behaviours	4	History of Smoking, Alcohol Abuse, Low/No Vigorous Activity, Current Smoker
ELSA	Adversity	1	Daily Discrimination
ELSA	Connections	7	History of Divorce, Lower Positive Interactions with Family, Negative Interactions with Family, Lower Positive Interactions with Children, Negative Interactions with Children, Negative Interactions with Friends, Never Married
ELSA	Psychological	2	Loneliness, Lower Life Satisfaction

Note: Sleep Problem, Mother education and father education are removed from ELSA due to their disproportional cross-distributions with deaths, which largely biased the death prediction.

Table S3: Risk Factors of Combined Datasets

Dataset	Domain	Variable Count	Variables
HRS+SHARE+ELSA	Demography	3	Age, Male, Foreign Born
HRS+SHARE+ELSA	Child-Adversity	0	
HRS+SHARE+ELSA	Socioeconomic	5	Lower Education, Wealth, History of Renting, History of Unemployment, Lower Occupational Status
HRS+SHARE+ELSA	Behaviours	3	Current Smoker, History of Smoking, Low/No Vigorous Activity
HRS+SHARE+ELSA	Adversity	0	
HRS+SHARE+ELSA	Connections	2	History of Divorce, Never Married
HRS+SHARE+ELSA	Psychological	0	
HRS+SHARE	Demography	3	Age, Male, Foreign Born
HRS+SHARE	Child-Adversity	3	Father Education , Mother Education , Father Occupational Status
HRS+SHARE	Socioeconomic	5	Lower Education, Wealth, History of Renting, History of Unemployment, Lower Occupational Status
HRS+SHARE	Behaviours	5	Current Smoker, History of Smoking, Low/No Moderate Activity, Sleep Problems, Low/No Vigorous Activity
HRS+SHARE	Adversity	1	Adulthood Psychosocial Adversity
HRS+SHARE	Connections	2	History of Divorce, Never Married
HRS+SHARE	Psychological	6	Hopelessness, Negative Affectivity, Lower Optimism, Perceptions of Obstacles, Pessimism, Lower Positive Affectivity
HRS+ELSA	Demography	3	Age, Male, Foreign Born
HRS+ELSA	Child-Adversity	0	
HRS+ELSA	Socioeconomic	8	Lower Education, Income, Lower Neighborhood Cohesion, Neighborhood Disorder, Wealth, History of Renting, History of Unemployment, Lower Occupational Status
HRS+ELSA	Behaviours	4	Alcohol Abuse, Current Smoker, History of Smoking, Low/No Vigorous Activity
HRS+ELSA	Adversity	1	Daily Discrimination
HRS+ELSA	Connections	7	Negative Interactions with Children, Negative Interactions with Family, Negative Interactions with Friends, Lower Positive Interactions with Children, Lower Positive Interactions with Family, History of Divorce, Never Married
HRS+ELSA	Psychological	2	Lower Life Satisfaction, Loneliness
SHARE + ELSA	Demography	3	Age, Male, Foreign Born
SHARE + ELSA	Child-Adversity	0	
SHARE + ELSA	Socioeconomic	5	Lower Education, Wealth, History of Renting, History of Unemployment, Lower Occupational Status
SHARE + ELSA	Behaviours	3	Current Smoker, History of Smoking, Low/No Vigorous Activity
SHARE + ELSA	Adversity	0	
SHARE + ELSA	Connections	2	History of Divorce, Never Married
SHARE + ELSA	Psychological	0	

6 Figures

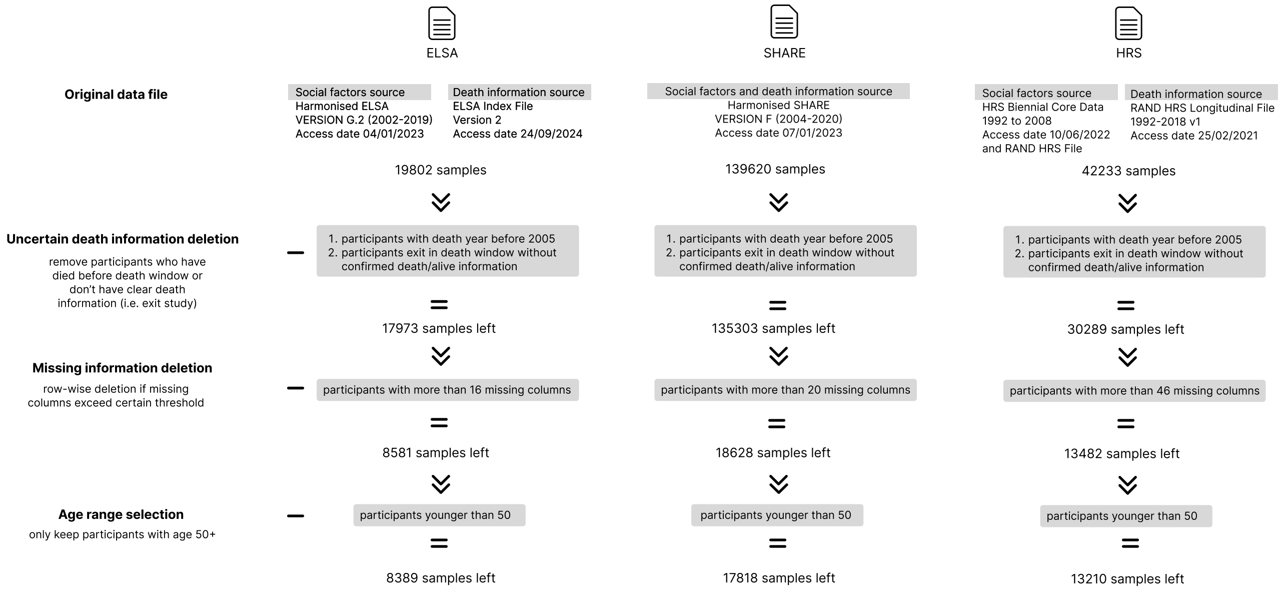

Figure S1: Data pruning process illustration for all datasets

Risk Factors	HRS	SHARE	ELSA	
History of Renting	0.30(0.46)	0.20(0.40)	0.20(0.40)	Socioeconomic
History of Food Insecurity	0.99(0.08)			
Wealth	437242.58(1116475.68)	230977.43(714183.45)	269109.34(388163.97)	
Lower Occupational Status	3.77(1.30)	3.63(1.35)	4.23(1.27)	
Recent Financial Difficulties	1.97(1.00)			
Lower Neighborhood Cohesion	2.47(1.35)		2.45(1.16)	
History of Medicaid	0.12(0.33)			
Income	69447.41(532410.00)		21071.13(19921.27)	
History of Unemployment	0.04(0.20)	0.05(0.21)	0.02(0.12)	
History of Food Stamps	0.12(0.32)			
Lower Neighborhood Safety	2.02(1.00)			
Neighborhood Disorder	3.60(1.65)		4.98(1.24)	
Lower Education	1.62(0.98)	4.36(1.43)	1.99(1.08)	
Adulthood Psychosocial Adversity	0.49(0.87)	0.05(0.33)		Adversity
Daily Discrimination	1.60(0.71)		1.55(0.54)	
Major Discrimination	5.48(1.90)			
Alcohol Abuse	0.07(0.25)		0.08(0.27)	Behaviours
History of Smoking	0.57(0.50)	0.51(0.50)	0.63(0.48)	
Current Smoker	0.12(0.32)	0.48(0.50)	0.18(0.39)	
Low/No Moderate Activity	0.70(0.46)	0.15(0.36)		
Low/No Vigorous Activity	0.77(0.42)	0.48(0.50)	0.72(0.45)	
Sleep Problems	0.28(0.45)	0.31(0.46)		
Childhood Psychosocial Adversities	0.36(0.61)			Child-Adversity
Father was Unemployed in Childhood	0.20(0.40)			
Relocated Homes in Childhood	0.18(0.39)			
Father Occupational Status	3.74(0.77)	4.45(1.33)		
Father Education	2.53(0.82)	4.99(1.13)		
Family Received Financial Help in Childhood	0.13(0.34)			
Mother Education	2.34(0.89)	5.39(0.81)		
Black	0.13(0.33)			Demography
Age	69.12(9.87)	63.96(9.61)	66.55(10.36)	
Male	0.41(0.49)	0.46(0.50)	0.44(0.50)	
Hispanic	0.08(0.27)			
Foreign Born	0.09(0.29)	0.11(0.31)	0.06(0.23)	
Lower Agreeableness	1.59(0.47)			Psychological
Lower Optimism	2.47(1.14)	2.27(0.67)		
Lower Sense of Mastery	2.23(1.10)			
Lower Life Satisfaction	2.81(1.38)		5.23(1.19)	
Lower Purpose in Life	2.40(0.92)			
Loneliness	1.48(0.54)		1.39(0.49)	
Lower Conscientiousness	1.91(0.51)			
Anger In	2.16(0.68)			
Lower Openness to Experiences	2.11(0.53)			
Perceptions of Obstacles	2.21(1.19)	1.95(0.79)		
Hopelessness	2.36(1.28)	-0.74(0.67)		
Lower Extroversion	1.86(0.50)			
Trait Anxiety	1.57(0.59)			
Lower Positive Affectivity	6.80(3.56)	2.26(0.69)		
Pessimism	2.59(1.28)	2.79(0.93)		
Lower Religiosity	1.97(1.37)			
Lower Neuroticism	2.53(0.66)			
Negative Affectivity	5.59(3.11)	1.61(0.47)		
Cynical Hostility	2.88(1.14)			
Anger Out	1.49(0.51)			
Negative Interactions with Friends	1.42(0.48)		1.56(0.51)	Connections
History of Divorce	0.18(0.38)	0.07(0.25)	0.09(0.29)	
Never Married	0.04(0.19)	0.05(0.21)	0.04(0.21)	
Negative Interactions with Children	1.71(0.62)		1.68(0.56)	
Lower Positive Interactions with Family	2.11(0.85)		2.15(0.84)	
Lower Positive Interactions with Children	1.73(0.70)		1.63(0.61)	
Negative Interactions with Family	1.57(0.61)		1.67(0.62)	

Figure S2: Risk factor inclusion and exclusion, mean value and standard deviation by dataset

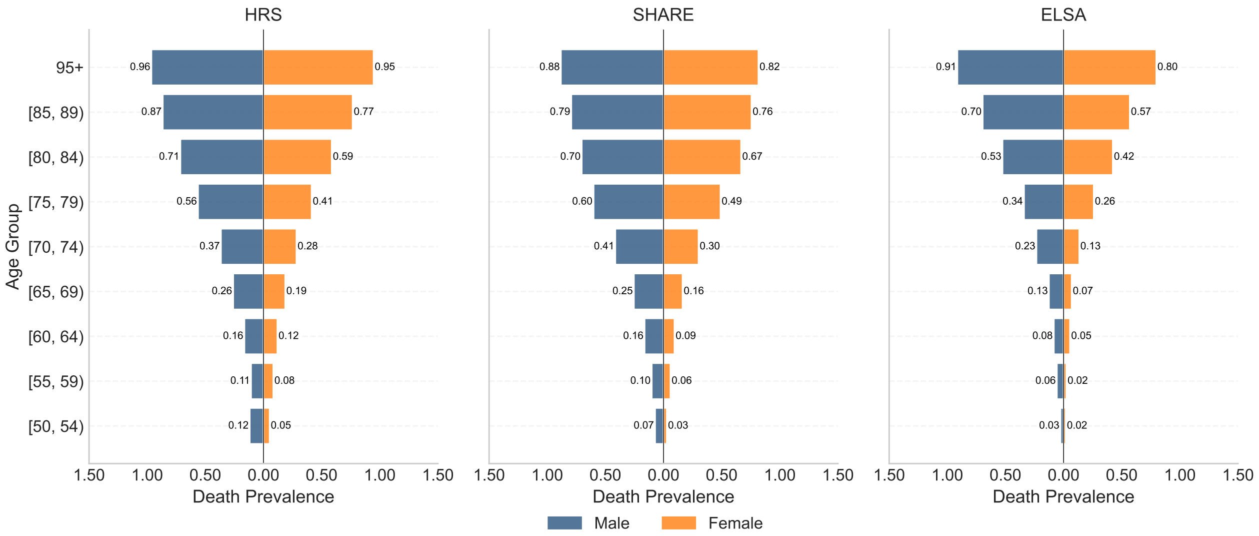

Figure S3: Death prevalence by age and gender for all datasets. For all datasets, we observe an increasing trend of death prevalence with increasing age groups, and for both genders. Compared to males, females of the same age have lower death prevalence in each dataset.

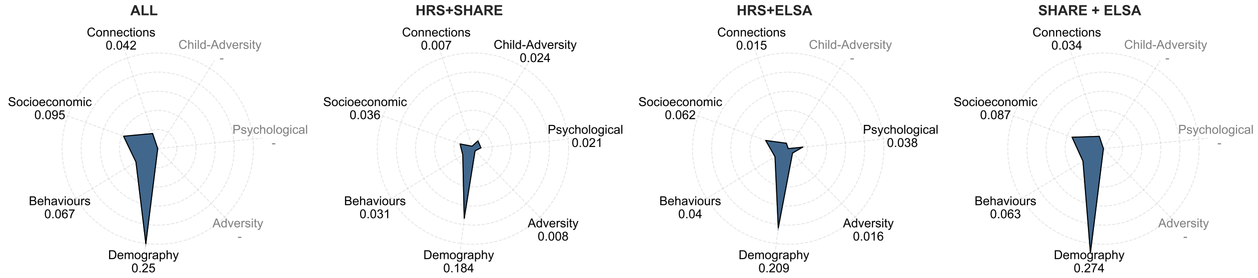

Figure S4: Domain contribution for all combined datasets. The observed pattern persists: demographic domain ranks the highest and then socioeconomic domain.

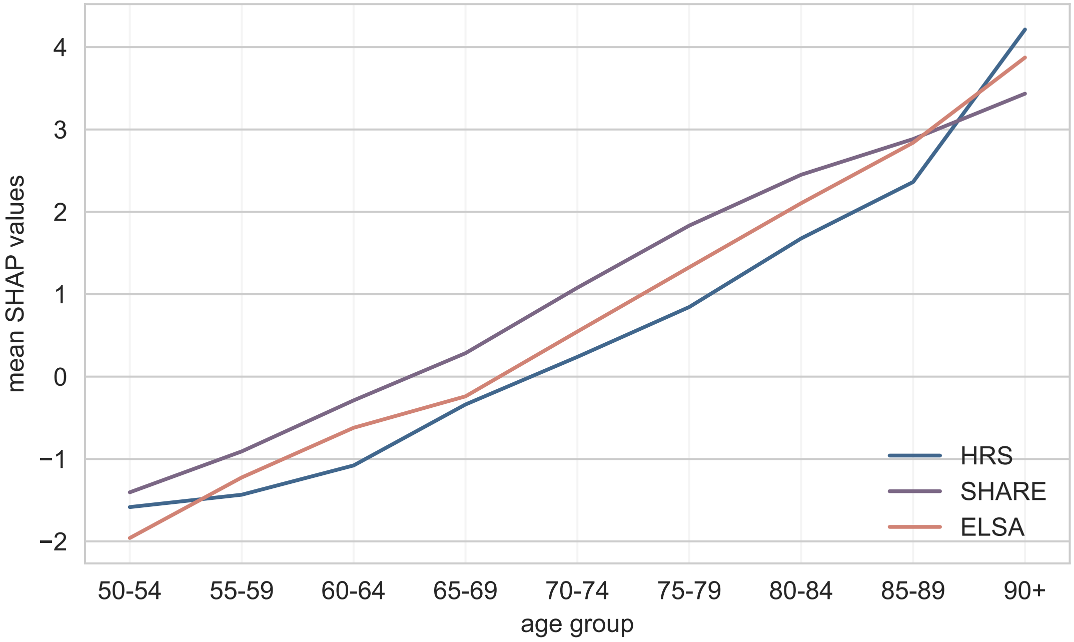

Figure S5: Mean SHAP Values of Age Across Different Age Groups. There is a clear and continuous upward trend of mean SHAP values for age across the three datasets, from younger to older age groups. There is a distinct pattern in mean SHAP values across the three datasets, transitioning from negative to positive as age increases. Negative mean SHAP values indicate that younger ages reduce the predicted risk of death relative to the average age of the sample, while positive values show that older ages increase predicted risk. The magnitude of SHAP values on either side of zero reflects how strongly age influences predictions, with larger absolute values indicating stronger relative influence, whether protective (negative SHAP) or risk-enhancing (positive SHAP).

References

[1] Mark J. van der Laan, Eric C. Polley, and Alan E. Hubbard. "Super Learner". In: *Statistical Applications in Genetics and Molecular Biology* 6.1 (Sept. 16, 2007). Publisher: De Gruyter. ISSN: 1544-6115. DOI: 10.2202/1544-6115.1309.

[2] Guolin Ke et al. "LightGBM: A Highly Efficient Gradient Boosting Decision Tree". In: *Advances in Neural Information Processing Systems*. Vol. 30. Curran Associates, Inc., 2017.

[3] Scott Lundberg, Gabriel Erion, and SuIn Lee. "Consistent Individualized Feature Attribution for Tree Ensembles". In: *arXiv:1802.03888 [cs, stat]* (Mar. 6, 2019). arXiv: 1802.03888.

[4] Benjamin W. Domingue et al. "The InterModel Vigorish (IMV) as a flexible and portable approach for quantifying predictive accuracy with binary outcomes". en. In: *PLOS ONE* 20.3 (Mar. 2025). Publisher: Public Library of Science, e0316491. ISSN: 1932-6203. DOI: 10.1371/journal.pone.0316491.

[5] Benjamin W. Domingue et al. "The InterModel Vigorish as a Lens for Understanding (and Quantifying) the Value of Item Response Models for Dichotomously Coded Items". eng. In: *Psychometrika* 89.3 (Sept. 2024), pp. 1034–1054. ISSN: 1860-0980. DOI: 10.1007/s11336-024-09977-2.
